# A Novel Method to Evaluate Ribosomal Performance in Cell-Free Protein Synthesis Systems

**DOI:** 10.1038/srep46753

**Published:** 2017-04-24

**Authors:** Noémie Kempf, Cristina Remes, Ralph Ledesch, Tina Züchner, Henning Höfig, Ilona Ritter, Alexandros Katranidis, Jörg Fitter

**Affiliations:** 1 Institute of Complex Systems ICS-5, Forschungszentrum Jülich, 52428 Jülich, Germany; 2 Physikalisches Institut (IA), RWTH Aachen, 52062 Aachen, Germany

## Abstract

Cell-free protein synthesis (CFPS) systems were designed to produce proteins with a minimal set of purified components, thus offering the possibility to follow translation as well as protein folding. In order to characterize the performance of the ribosomes in such a system, it is crucial to separately quantify the two main components of productivity, namely the fraction of active ribosomes and the number of synthesizing cycles. Here, we provide a direct and highly reliable measure of ribosomal activity in any given CFPS system, introducing an enhanced-arrest peptide variant. We observe an almost complete stalling of ribosomes that produce GFPem (~95%), as determined by common centrifugation techniques and fluorescence correlation spectroscopy (FCS). Moreover, we thoroughly study the effect of different ribosomal modifications independently on activity and number of synthesizing cycles. Finally, employing two-colour coincidence detection and two-colour colocalisation microscopy, we demonstrate real-time access to key productivity parameters with minimal sample consumption on a single ribosome level.

Cell-free protein synthesis (CFPS) was established many decades ago, based on the knowledge that cell integrity is not required to express proteins from genes^1^. In its simplest form, translation can be accomplished with a crude lysate from a given organism that provides the translational machinery, accessory enzymes, tRNAs and further factors. In most applications, coupled systems, where a combined transcription and translation configuration is used, exhibit reasonable protein yields and are easy and fast to operate^2^,^3^. Since CFPS takes place without any cell wall barriers, it offers a large flexibility and allows direct molecular manipulations of the transcription and translation factors. This includes the introduction of exogenous modified components (e.g. labelled or biotinylated ribosomes), frequently required for fluorescence-based studies investigating transcription, translation or cotranslational protein folding^4^,^5^,^6^,^7^,^8^,^9^,^10^.

Despite the promising capabilities of CFPS systems, the fast consumption of the high energy phosphate pool and the accumulation of side products inhibit protein synthesis and lead to relatively short reaction times^11^. A number of improvements over the years, regarding energy supply and small molecule metabolism, addressed partly these issues and increased significantly the performance of the CFPS systems^2^. Nevertheless, the effect of the ribosomes on the performance is still not fully studied. To this aim, polysomes profile analysis^12^ and real-time assays based on green fluorescence protein (GFP) synthesis^13^,^14^ have been developed and led to the characterization of CFPS expression yields under different conditions.

Here, we present a new strategy to measure on-the-fly the active fraction of ribosomes with high reliability in any given CFPS system. Our method has the advantage over previous studies that it can be used without the need of any labour-intensive assays or additional pre-treatment of the sample. More specifically, we produced a fast maturating GFP variant using exogenous modified ribosomes in a CFPS system. Our method is based on the possibility to reach an almost complete stalling of the ribosomes. To this aim we quantitatively compared the stalling efficiency of different short arrest peptides (AP) that are well known to interact with the ribosomal exit tunnel and arrest the translation continuation^15^,^16^,^17^, by changing the geometry of the peptidyl transferase centre (PTC), thus inhibiting peptide bond formation^18^,^19^. Additionally, we could quantify the impact of ribosomal modifications on the productivity, separately addressing the fraction of active ribosomes and the number of synthesizing cycles. Finally, in addition to the conventional centrifugation techniques, we confirmed the reliability of the method with fluorescence correlation spectroscopy (FCS) and extended the study to the single ribosome level, both in solution by two-colour coincidence detection (TCCD)^20^ and on glass surfaces by two-colour colocalisation microscopy.

## Results

### Ribosome stalling during nascent chain synthesis

**Figure 1:**
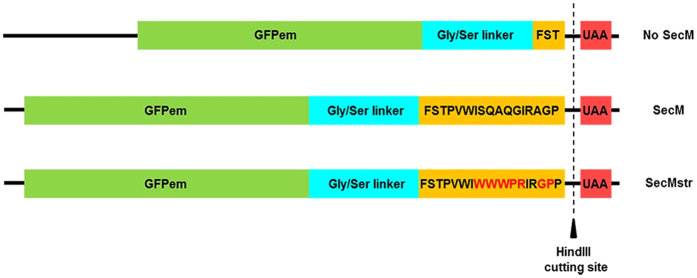
Cartoon of the constructs used. The gene of GFPem was followed by a 30-residue linker rich in Gly and Ser, the 17-residue SecM AP or SecMstr AP and a stop codon. The mutated amino acids of SecMstr AP are shown in red letters. The control construct is missing the last 14 residues of the SecM moiety. All constructs could be linearized removing at the same time the stop codon.

Initially, we compared the degree of ribosome stalling during nascent chain synthesis for three different constructs. The first construct included the widely used but rather weakly stalling 17-residue SecM AP from *Escherichia coli*^5^,^15^,^21^,^22^,^23^, while the second a SecM AP variant further referred to as SecM strong (SecMstr), which we constructed following suggestions described in a recent study on enhanced-arrest variants^24^. Finally a third construct without any arrest sequence was used as a control. All three constructs also contained the sequence of GFPem and a C-terminal extension with a 30-residue linker rich in Gly and Ser followed by either the SecM AP or the SecMstr AP (Fig. 1 and Supplementary Fig. S1). Thanks to the combination of Gly/Ser linker and the AP, the C-terminal extension was able to span the full length of the ribosomal tunnel allowing the complete GFPem to emerge and fold while still being attached to the ribosome. The control was missing the last 14 residues of the arrest sequence, thus eliminating its stalling effect on the translating ribosome (Fig. 1 and Supplementary Fig. S1). We also investigated the use of linear DNA constructs as another common method for ribosome stalling^6^,^14^. To this end, all constructs could be readily linearized, concomitantly removing the stop codon.

GFPem was expressed *in vitro* using a ribosome-depleted CFPS system under non-limiting conditions (see Methods and Supplementary Fig. S2). After a 2 h reaction, stalling was verified with two independent methods; co-precipitation and FCS measurements. In the co-precipitation experiments, the sample was centrifuged through a sucrose cushion following a previously established protocol^14^. Since only the heaviest particles, i.e. the ribosomes, were able to pass through the sucrose cushion, the amount of GFPem found in the pellet represented the fraction that remained bound after synthesis and thus co-precipitated with the ribosomes. On the contrary, the amount of GFPem present in the supernatant corresponded to the released protein.

**Figure 2:**
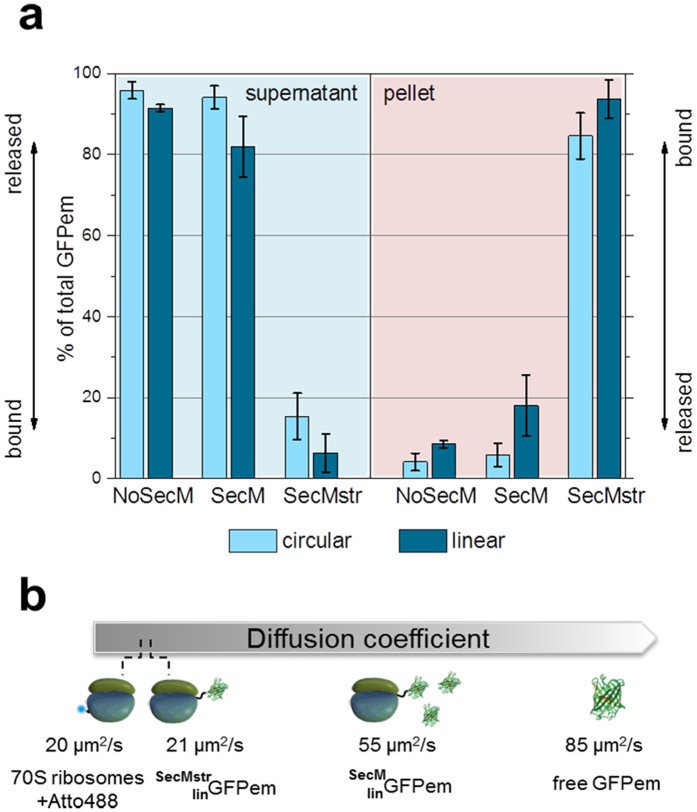
Stalling efficiency of different constructs as determined by co-precipitation. **(a)** Relative amount of GFPem (after 2 h CFPS reaction) in supernatant (cyan background) and pellet (red background) after centrifugation through sucrose for circular (light blue bars) and linear (dark blue bars) constructs. High amounts of GFPem in the supernatant (No SecM, SecM) suggest substantial release of the nascent chain. On the contrary, high amounts in the pellet (SecMstr) indicate efficient stalling. The experiments were carried out at least 3 times for each condition (biological replications). (**b)** Corrected values of diffusion coefficients D_0_ (see Supplementary Fig. S1) measured in FCS for 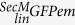 and 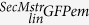 without centrifugation (after 2 h CFPS reaction). Corresponding values for free GFPem and labelled 70S ribosomes are given for comparison. The intermediate value for 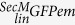 reveals partial release, while the ribosome-like diffusion of 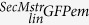 indicates efficient stalling.

We studied the effect of the linearization of our constructs by comparing the quantities of GFPem present in the pellet and in the supernatant. We observed that for all three constructs, the linearization only brought a minor increase of the stalling efficiency, indicating that it was rather insufficient by itself to prevent the release of the GFPem (Fig. 2(a), compare light blue and dark blue bars). Nevertheless, since better stalling was obtained after linearization, the following descriptions will only consider the linear constructs. As expected, without any AP, the vast majority of GFPem was released from the ribosomes (>95% of free GFPem) (Fig. 2(a), cyan background). Even the presence of the SecM AP did not lead to a significant increase of the bound fraction of GFPem (<20% of bound GFPem), in contrast to the enhanced-arrest variant SecMstr, which showed an impressive increase of the stalling efficiency (>90% of bound GFPem) (Fig. 2(a), red background). The high stalling efficiency of the SecMstr AP compared to the SecM AP was also confirmed by addition of Kasugamycin (Ksg) to the CFPS system. Ksg known to block translation initiation by preventing the association of the ribosomal subunits, has no effect on translating or stalled 70S ribosomes^25^,^26^,^27^ (see Supplementary Text and Supplementary Fig. S3).

To further investigate the AP-dependent ribosome stalling, we employed Fluorescence Correlation Spectroscopy (FCS). Directly after the CFPS reaction we compared the diffusion coefficients D_0_ of GFPem synthesised with both APs, to the measured values of free GFPem and labelled 70S ribosomes (Fig. 2(b) and Supplementary Fig. S4). The diffusion of ribosomes (D_0_ = 20 μm^2^/s) was significantly slower than the one of free GFPem (D_0_ = 85 μm^2^/s), thus by monitoring the diffusion of GFPem, synthesised using the linear constructs with the SecM AP (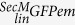) or the SecMstr AP (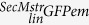) we could directly observe the stalling efficiency. In good agreement with the previous co-precipitation assay, 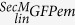 showed an intermediate diffusion (D_0_ = 55 μm^2^/s) suggesting that it was largely released and freely diffusing. On the contrary, a slow diffusion (D_0_ = 21 μm^2^/s), very similar to the diffusion of ribosomes, was observed for the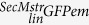 indicating an almost complete binding (Fig. 2(b) and Supplementary Fig. S4). Additionally, we measured the pellet after co-precipitation, which showed ribosome-like diffusion of GFPem regardless of the used construct. Hence, FCS measurements confirmed that released and bound GFPem could be efficiently separated by centrifugation and in addition that the GFPem remained physically bound even after centrifugation (Supplementary Fig. S4).

Interpreting the co-precipitation and the FCS results we concluded that the SecMstr AP stalled almost completely the ribosomal machinery and by keeping the GFPem bound, obstructed at the same time the initiation of a new cycle of synthesis. Therefore, the active fraction of ribosomes in a CFPS reaction mix could be easily identified by quantifying the amount of synthesised GFPem molecules, without any further selection or treatment of the sample. This is especially advantageous when performing *in situ* measurements, where any treatment prior to the reaction is by definition not possible.

### Effect of ribosomal modifications on activity and productivity

In CFPS only a fraction of the ribosomes is active, nevertheless each active ribosome can undergo several cycles of synthesis. Therefore, we determined productivity as the total amount of GFPem that was produced, considering the active fraction of ribosomes and the number of synthesizing cycles they would undergo. In the case of 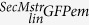, since only a single productive cycle could take place per active ribosome due to the high efficiency of the stalling mechanism, the amount of produced GFPem was directly revealing the fraction of active ribosomes. Here, we investigated separately the impact on both the activity and the productivity, caused by biotinylation and fluorescent labelling^6^,^28^,^29^,^30^,^31^,^32^, two commonly used ribosomal modifications that enable direct monitoring and if desired, immobilization of ribosomes in single-molecule experiments.

**Figure 3:**
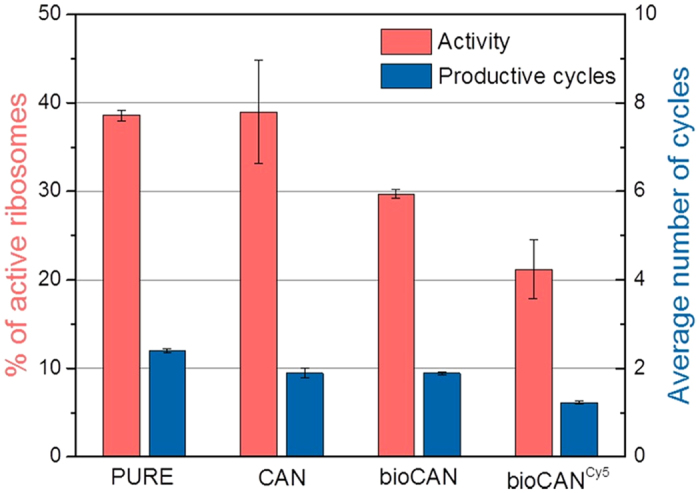
Activity and number of synthesizing cycles for different ribosomal modifications. (from left to right) PURE System ribosomes; CAN20/12E ribosomes; biotinylated CAN20/12E ribosomes; biotinylated CAN20/12E ribosomes labelled with Cy5. Activity (red bars) and number of productive cycles (blue bars) were determined by expressing and measuring the concentration of 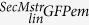 (efficient stalling, only one productive cycle) and 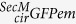 (almost no stalling, constant activity assumed), respectively (after 2 h CFPS reaction). Activity drops with each modification step, while the number of synthesizing cycles remains mainly constant at a low level. The experiments were carried out at least 3 times for each condition (biological replications) in triplicates (technical replications).

First, we examined the effect of these modifications on the ribosomal activity by comparing the overall production of 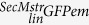. To this aim, we compared our “home-made” CAN20/12E ribosomes (CAN) with the commercially available PURE system ribosomes and observed a fraction of ~40% active unmodified ribosomes in both cases (Fig. 3, red bars). These data indicate that the quality of CAN ribosomes are comparable to commercially available ones and thus represent a good reference for further studies. Afterwards, we investigated the effect of biotinylation and fluorescent labelling. For this purpose, CAN ribosomes were biotinylated (bioCAN) with a single biotin at the uL4 ribosomal protein or both biotinylated and labelled with ~6 Cy5 dyes (bioCAN^Cy5^) (see Methods). Our data showed that biotinylation reduced the active fraction to ~30% and upon additional fluorescent labelling activity dropped to ~20% (Fig. 3, red bars). Interestingly, a second batch of the same ribosomes that underwent an identical labelling procedure in parallel but without addition of the dye, showed only a slightly lower decrease of the active fraction, revealing that mostly the treatment and much less the presence of the dye reduced the ribosomal activity (Supplementary Fig. S5).

In a next step, we investigated the effect of the modified ribosomes on the productivity and consequently on the number of synthesizing cycles. For this purpose, we used the circular construct of GFPem containing the SecM AP (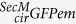), where stalling was mostly inefficient (Fig. 2a) and considered the final amount of synthesised GFPem as the maximum that our system without stalling could achieve. Then, by considering the previously measured activity, we could calculate the number of productive cycles. As shown, the active CAN and bioCAN ribosomes underwent an average of merely two synthesizing cycles, while the bioCAN^Cy5^ ribosomes slightly more than one cycle on average (Fig. 3, blue bars) suggesting that, the biotinylation of the ribosomes affected only the activity, but not the number of active cycles. In contrast, the additional labelling treatment affected the total productivity, both by reducing the fraction of active ribosomes and the number of productive cycles.

In order to establish alternative methodological approaches and to verify our above described findings we used the modified ribosomes to perform studies on single ribosome level. Therefore, we employed two fluorescence techniques, namely two-colour coincidence detection (TCCD)^20^ and two-colour colocalisation microscopy to study individual bioCAN^Cy5^ ribosomes in solution and on a glass surface, respectively. The red labelled ribosomes synthesised 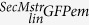 using a CFPS reaction mix and were afterwards diluted down to pM concentration suitable for single-molecule measurements. With both techniques, we were able to detect single ribosomes and GFPem molecules as well as their synchronous occurrence, without any centrifugation steps or additional assays.

In TCCD, simultaneous detection of Cy5 and GFPem fluorescence evidenced the diffusion of single ribosomes with a bound synthesised protein. The active fraction of ribosomes (~20%, Table 1) was calculated by taking the number of coinciding events out of all detected red fluorescent ribosomes, and was in good agreement with the ensemble measurements. Similarly, the stalling efficiency (~85%, Table 1) was calculated by taking the fraction of coinciding events out of all detected green fluorescent GFPem molecules (see Methods). This value, lower than the ensemble measurement result (~94%) was shown to be influenced by background bursts coming from the labelled ribosomes solution (see Supplementary Text and Supplementary Table S1).

**Figure 4:**
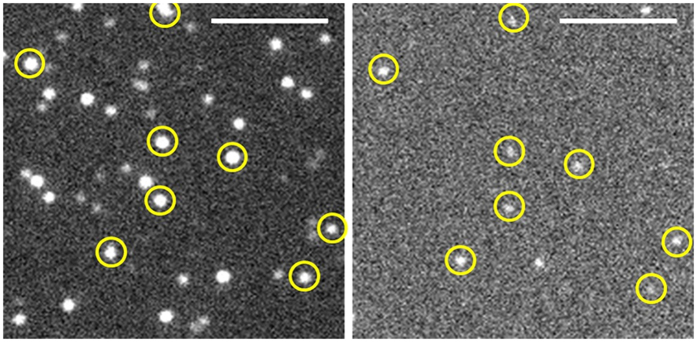
Two-colour colocalisation microscopy. Surface adsorbed 70S bioCAN^Cy5^ ribosomes (left) and 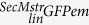 (right) images were acquired simultaneously at the same sample area. Colocalised ribosome (8 out of 49) and GFPem (8 out of 10) signals are marked (yellow circles). Scalebar: 5 μm.

In two-colour colocalisation microscopy, ribosomes with bound GFPem molecules were recognized as signals from both channels at the same surface position (Fig. 4). We determined the fraction of active ribosomes (~17%, Table 1) as the percentage of colocalised signals out of all immobilized ribosomes, also being in good agreement with ensemble and TCCD measurements. Likewise, the stalling efficiency (~85%, Table 1) was determined by the fraction of colocalised signals out of all GFPem molecules on the surface (see Methods). Finally, the data collected from the different approaches presented in this work, clearly demonstrate that the values for activity and stalling determined on single ribosome level are comparable to a large extent with the ensemble observations.

## Discussion

Here we address in more depth the question of the ribosomal productivity in a CFPS system by disentangling its two contributions; the active fraction of ribosomes and the number of synthesizing cycles. In this respect, an efficient stalling of the ribosomes was essential. Our data showed that only the introduction of the SecMstr AP could suppress the release of the nascent chain satisfyingly, while mere linearization and addition of the widely used SecM AP were mostly inefficient. This may not be so surprising, since it was previously reported that in the absence of any force arrest release of SecM-stalled ribosomes takes place with a half-time of ~42 min^33^. In addition, recent studies also pointed out that the SecM-stalled nascent chain can be released due to the mechanical force applied by its cotranslational folding^33^,^34^. Thus, it is quite probable that the stronger SecM AP-variant can withstand the applied force due to the folding of GFPem much better than the weak SecM AP. The increased stalling efficiency of the SecMstr AP could be partly attributed to the incorporation of the two consecutive Pro residues of its sequence. Peptide bond formation between a pair of Pro takes place at a very low rate to begin with^35^,^36^. Adding to that the coexistence of two residues with limited degree of freedom in the ribosomal peptidyl transferase centre could well inhibit any further amino acid incorporation.

Following the successful ribosome stalling, we studied the productivity of ribosomes in a CFPS system. More specifically, we separately showed the impact on activity and number of productive cycles caused by two modifications commonly used in fluorescence and single-molecule studies; biotinylation and fluorescent labelling. Our results indicated that activity was mainly affected by the treatment of the sample rather than the incorporated modifications (biotin and label) (Fig. 2). The reduced activity of the biotinylated ribosomes in comparison to the non-biotinylated ribosomes was due to a late ribosome isolation during the cell growth and not caused by the attached biotin residue itself. Indeed, harvesting the cells in the early mid-log phase is optimal for the activity of the isolated ribosomes in contrast to the late-log phase where activity drops^37^,^38^. It was rather straightforward to harvest the cells in the early mid-log phase when isolating the unmodified CAN ribosomes. On the contrary, for the bioCAN ribosomes an additional induction of the cells was necessary for the *in vivo* biotinylation, shifting the harvesting towards the late-log phase. Efforts to harvest the cells at an earlier stage indeed showed an increased activity, strongly suggesting that the harvesting time was the main reason of the activity drop (data not shown). Similarly, our data showed that the labelling procedure had the main impact on the reduced activity, while the presence of the dye on the ribosomes only contributed to a lesser extent (Supplementary Fig. S5). Nevertheless it should be mentioned that by using alternative labelling procedures, a different impact on the activity could well be observed.

Furthermore, by knowing the fraction of active ribosomes, we were able to calculate the number of productive cycles they could undergo in a CFPS reaction, in the absence of stalling. It appeared that the number of productive cycles was considerably low, both for modified and unmodified ribosomes. This low number of cycles and consequently the weak productivity can be mostly attributed to the limitations of the CFPS system, namely accumulation of inhibitory by-products and depletion of energy resources, which constitute a major problem in the field. The use of a different CFPS system did not result in higher number of productive cycles (data not shown). Nevertheless, employing a Continuous-Exchange Cell-Free (CECF) system^11^,^39^, where an exchange of substrates and by-products takes place, raised the productivity of the sample thanks to the higher number of productive cycles (~15 cycles) completed by the active ribosomes (Supplementary Table S2). Apparently, more productive CFPS systems were reported^12^. However, the focus of this study was to measure reliably the active fraction of ribosomes and the number of synthesizing cycles in any given system. This could be potentially used to determine how productive a given CFPS system is and help monitor targeted improvements of the system. Our approach offers to address ribosomal activity and system conditions separately: keeping the system parameters (buffer conditions, added components etc.) fixed, the activity of differently prepared (time of harvesting, further treatment procedures etc.) or modified (biotinylated, labelled etc.) ribosomes can be directly compared. Thus, the impact of various parameters can be quantified and the productivity of CFPS systems optimized through higher active ribosome fractions. Once the ribosomal activity has been determined, the influence of different synthesis conditions (buffer composition, synthesis time etc.) and addition of exogenous components (chaperones, initiation factors, elongation factors etc.) in CFPS systems can be monitored for a given ribosome “type”. In this case, CFPS system productivity can be optimized through increased productive cycles of the active ribosomes. As mentioned above, a comparison between single-molecule techniques and co-precipitation experiments revealed equivalent values regarding activity (Table 1), while the values of the stalling efficiency obtained from single-molecule studies were systematically lower. It cannot be excluded that at these extremely low concentrations possible dissociation of a small fraction of the ribosomes-nascent chain complexes affect the single-molecule data leading to these discrepancies. However, despite the deviations between the single-molecule and ensemble measurements, the general reliability and consistency of each individual method was confirmed. Single-molecule techniques allow therefore the direct determination of valuable parameters characterizing the performance of CFPS systems without the need of further purification steps and by the use of low sample quantities.

In summary, we quantified the performance of ribosomes with an approach that is applicable to any CFPS system, providing that factors rescuing ribosomes from stalling are completely absent or blocked (elongation factor P, tmRNA etc.). More specifically (i) we used an enhanced AP variant and created an almost absolute stalling of the ribosomes (>95%), providing a direct and highly reliable measure of the active ribosomal fraction without the need for further treatment, (ii) we succeeded to disentangle and study independently the activity and the number of synthesizing cycles of modified ribosomes, which could assist in a more targeted optimization of the CFPS systems and finally (iii) we demonstrated that single-molecule techniques are suitable to measure reliable and consistent parameters characterizing the CFPS system. Further interesting applications of such techniques, which require very small sample amounts, would be to study cell-free synthesis in crowding conditions (microgels) or in confined environments (CFPS systems encapsulated in vesicles)^40^,^41^ or follow protein synthesis during *in situ* experiments of single surface-tethered ribosomes over extended time periods. Apart from the possibility to monitor, characterize and optimize the activity and productivity of ribosomes in a CFPS system, the combination of the enhanced stalling efficiency with single-molecule measurements offers a broader experimental approach in the field of transcription, translation and cotranslational protein folding.

## Methods

All samples were prepared in RNase-free environment. Surfaces were cleaned with RNaseZAP (Sigma-Aldrich, St. Louis, USA) and RNase-free pipette tips (Biozym Scientific GmbH, Hessisch Oldendorf, Germany) were used.

### Plasmid

The plasmid pRSET/EmGFP (Thermo Fisher Scientific, Waltham, USA), containing the gene of GFP variant Emerald (GFPem: F64L, S65T, S72A, N149K, M153T, I167T)^42^, was modified by adding a 47-residue C-terminal extension, between the XhoI and HindIII sites. The extension consists of a region rich in Gly and Ser followed by the SecM (FSTPVWISQAQGIRAGP)^21^ or the SecMstr AP (FSTPVWIWWWPRIRGPP), where the underlined residues were changed according to reference^24^. The SecM AP induces translational arrest, by shifting the linkage between the nascent chain and the peptidyl-tRNA by 2 Å^18^,^19^. A construct missing the last 14 residues of the AP was used as control. The plasmid could be linearized in all cases upon digestion with the restriction endonuclease HindIII, removing at the same time the stop codon.

### Ribosomes

To avoid RNase and protease contamination, RNase inhibitors (SUPERase• In™ RNase Inhibitor (20 U/μL), Thermo Fisher Scientific, Waltham, USA) and protease inhibitors (cOmplete Protease Inhibitor Tablets, EDTA Free, Roche, Basel, Switzerland) were added to all buffers used for isolation of ribosomes, according to the manufacturer’s instructions.

### Biotinylation

The RNase deficient *E. coli* K-12 strain CAN20/12E (RNase BN^−^, II^−^, D^−^, I^−^)^43^ was transformed with two plasmids. The pBirAcm plasmid contained the birA gene overexpressing biotin ligase and the pAN5 plasmid the ribosomal protein uL4 fused to an N-terminal AviTag (14-mer peptide GLNDIFEAQKIEWH) (Avidity, Aurora, USA). The biotin ligase catalyzes transfer of a single biotin molecule specifically at the lysine of the 14-residue AviTag^44^. Upon induction with 1 mM IPTG at A_600_ = 0.4 and addition of 50 μM biotin, *in vivo* biotinylation took place by the biotin ligase. Cells were grown for an additional 1 h at 37 °C and incubated on ice for 1 h before harvesting.

### Isolation

Biotinylated ribosomes were isolated by sucrose gradient centrifugation using a zonal rotor as previously described^38^ and resuspended in Tico buffer [20 mM Hepes-KOH (pH 7.6 at 0 °C), 10 mM magnesium acetate, 30 mM ammonium acetate, 4 mM β-mercaptoethanol]. Activity was checked by synthesizing GFPem using an *in vitro* transcription/translation system (PURExpress Δ ribosomes, NEB #E3313, New England Biolabs, Ipswich, USA). GFPem fluorescence was monitored in a QM-7 spectrofluorometer (Photon Technology International, Birmingham, USA).

### Labelling

Biotinylated ribosomes reacted with a Cy5-NHS-ester (GE Healthcare Life Sciences, Little Chalfont, UK) or Atto488-NHS-ester functionalized dye (Atto-Tec GmbH, Siegen, Germany) in labelling buffer [50 mM Hepes-KOH (pH 7.5), 10 mM MgCl_2_, 100 mM KCl] for 20 min at 37 °C, using a 20-fold excess of dye to minimize the unlabelled fraction of ribosomes. The excess of dye was removed by pelleting the ribosomes through a 1.1 M sucrose cushion. After resuspending the pellet in Tico buffer, aggregates were removed by centrifugation and the supernatant was used for further experiments. The concentration of Cy5 and ribosomes was determined spectroscopically in a NanoDrop spectrophotometer (Thermo Fisher Scientific, Waltham, USA) using the absorption coefficients ε_cy5_ = 2.5 × 10^5^ M^−1^ cm^−1^ and ε_ribos_ = 4.2 × 10^7^ M^−1^ cm^−1^, respectively. The label ratio was calculated to be ~6 Cy5 dyes per ribosome.

### Cell-free protein synthesis

The cell-free transcription/translation system used in this study was the PURE system^45^ without ribosomes (PURExpress Δ ribosomes, NEB #E3313, New England Biolabs, Ipswich, USA). A cell-extract-based fractionated transcription/translation system (RiNA GmbH, Berlin, Germany) was also used for comparison (data not shown). Reactions were performed according to the manufacturer’s protocol containing 500 nM of ribosomes and 5.5 nM of circular or linear plasmid. A higher concentration of 20 nM linear plasmid was also used to check whether the reaction is performed under limiting conditions (Supplementary Fig. S2). Since tmRNA can induce the ribosome-nascent chain complex dissociation^46^,^47^, 5 nM of anti-sense tmRNA (AS-tmRNA)^48^, complementary to the tmRNA sequence was added, whenever the linear plasmid was used, in order to suppress its function. The concentration of ribosomes was calculated by measuring the absorption at 260 nm in a NanoDrop spectrophotometer (Thermo Fisher Scientific, Waltham, USA) and using an absorption coefficient ε_ribos_ = 4.2 × 10^7^ M^−1^ cm^−1^.

For the co-precipitation experiments, the reaction mixtures were diluted and layered onto 1.1 M sucrose in Tico buffer and centrifuged at 110 000 × g for 2.5 h. The pellet was resuspended in Tico buffer and the concentration of the ribosomes was calculated by measuring absorption at 260 nm. The concentration of GFPem in the supernatant and in the pellet was determined spectroscopically as mentioned below.

### Ribosomal productivity measurements and analysis

Fluorescence spectra of synthesised GFPem were recorded over time at 37 °C on a QM-7 spectrofluorometer (Photon Technology International, Birmingham, USA) using 3 mm path length quartz cuvettes (105.251-QS, Hellma, Mühlheim, Germany). All spectra were corrected for background intensities by subtracting the spectra of the solvent measured under identical conditions. In addition, a manufacturer-provided correction accounted for the detection efficiency function. The measurements were carried out at least in triplicates. The samples were excited at λ_ex_ = 465 nm and the emission spectra were recorded between 480–650 nm. GFPem concentrations were determined from the measured fluorescence using a calibration curve with known GFPem concentrations measured under the same conditions. The calculated GFPem concentration was normalized to the concentration of ribosomes.

### Confocal fluorescence detection

Fluorescence Correlation Spectroscopy (FCS) and single-molecule two-colour coincidence detection (TCCD) measurements^20^ of freely diffusing fluorescent species were performed with a MicroTime 200 confocal microscope (PicoQuant, Berlin, Germany). The excitation light from pulsed lasers at 485 nm and 633 nm (LDH-D-C 485 and LDH-D-C 640, PicoQuant, Berlin, Germany) was focused with a water immersion objective (UplanSApo, 60×, NA 1.2, Olympus Deutschland, Hamburg, Germany) 15 μm above the glass surface. The same objective was used for fluorescence collection in combination with a dual-band dichroic mirror (XF2401, Omega Optical, Brattleboro, USA). After focusing through a 30 μm (FCS) or 75 μm (TCCD) pinhole, the fluorescence was split into a blue and red detection channel by another dichroic mirror (T600lpxr, Chroma Technology Corp, Bellows Falls, USA). 50/50 beam splitter cubes (Olympus Deutschland, Hamburg, Germany) and appropriate emission filters (blue: 530/55 Semrock, Rochester, USA, red: ET685/80 M, Chroma Technology Corp, Bellows Falls, USA) enabled detection on two single-photon avalanche diodes for each channel (blue: τ-SPAD, PicoQuant, Berlin, Germany, red: SPCM-CD3077-H and SPCM-AQR-14, Perkin-Elmer Inc., Waltham, USA). The arrival time of each photon was recorded with a time-correlated single-photon counting module (PicoHarp300, PicoQuant, Berlin, Germany).

For FCS, nM concentrations were used, while pM dilutions were fixed for TCCD, resulting in average numbers of 0.01 ribosomes in the effective volume. FCS analysis was performed using the SymPhoTime64 software (PicoQuant, Berlin, Germany). For TCCD, we applied pulsed interleaved excitation (PIE)^49^ to identify two-colour fluorescent species and single-colour events. Data analysis was done with self-written Matlab routines (Mathworks, Natick, USA). In short, bursts were selected through the inter-photon distance and coincidence analysis was similar to the approach used by Li *et al*.^50^.

### Surface adsorption of ribosomes

Home-built sample chambers consisted of a pair of plasma cleaned (Diener Electronic, Ebhausen, Germany) high precision glass slides (170 ± 5 μm, No. 1.5 H, Marienfeld Superior, Lauda-Königshofen, Germany) fixed together by double-sided tape forming a channel. 50 pM of ribosomes were added and thoroughly washed with Tico buffer.

### Two-colour colocalisation microscopy

Surface imaging was performed with an inverted microscope (Olympus IX-81, Olympus Corp., Shinjuku, Japan) in widefield illumination mode. For excitation, lasers at 488 nm and 639 nm (Sapphire 488-200 and Obis 637-140, Coherent Inc., Santa Clara, USA) were focused into the back-focal plane of an oil-immersion objective (Olympus UApoN, 100×, NA = 1.49, Olympus Corp., Shinjuku, Japan). The fluorescent light was collected with the same objective and passed through a dual-band dichroic mirror as well as a dual-band emission filter (ZT488/640rpc and ZET488/640 m, Chroma Technology Corp, Bellows Falls, USA). Simultaneous dual-colour imaging was implemented with an image splitter (Optosplit II, Cairn Research Ltd, Faversham, UK, dichroic mirror DCLP 600DCXR, Omega Optical Inc, Brattleboro, USA) on an EMCCD camera (Andor iXon Ultra 888, Andor Technology Ltd., Belfast, UK)) with an additional emission filter in the red channel (ET655lp, Omega Optical Inc, Brattleboro, USA).

An oxygen scavenger solution was added (20 U/ml glucose oxidase, 200 U/ml catalase, 1% w/v glucose in Tico buffer) and images were acquired for 1.5 s at 10 mW (blue) and 7.5 mW (red) excitation power. For data analysis, we used self-written Matlab routines (Mathworks, Natick, USA). In both channels, individual and colocalised signals (of multi-labelled ribosomes and single GFPem molecules) were identified taking into account chromatic aberrations.

### Activity and stalling efficiency calculations

For TCCD and colocalisation microscopy the activity is given by:


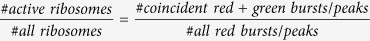


Similarly, the stalling efficiency is given by:


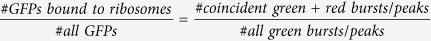


## Additional Information

**How to cite this article:** Kempf, N. *et al*. A Novel Method to Evaluate Ribosomal Performance in Cell-Free Protein Synthesis Systems. *Sci. Rep.* **7**, 46753; doi: 10.1038/srep46753 (2017).

**Publisher's note:** Springer Nature remains neutral with regard to jurisdictional claims in published maps and institutional affiliations.

## Supplementary Material

Supplementary Information

## Figures and Tables

**Table 1:** Activity and stalling efficiency of ribosomes expressing 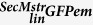 .

Techniques	Activity [%]	Stalling [%]
Co-precipitation	21.2 ± 3.8	93.6 ± 4.7
Two-colour coincidence detection	20.3 ± 7.7	84.7 ± 6.5
Two-colour colocalisation microscopy	17.4 ± 3.3	85.0 ± 5.9

Activity and stalling efficiency were measured with co-precipitation, TCCD (analysis of >28 000 ribosomes) and two-colour colocalisation microscopy (analysis of >12 500 ribosomes). For each technique, a minimum of three independent experiments was performed. The given errors are absolute standard deviations calculated from the repetitive measurements.
