# Supplementary material for: A Novel Method to Evaluate Ribosomal Performance in Cell-Free Protein Synthesis Systems
Source: Sci Rep. 2017 Apr 24;7:46753. doi: 10.1038/srep46753 (PMC5402277; doi:10.1038/srep46753)
Supplement: Supplementary Information [file srep46753-s1.pdf]

Supplementary Information

A Novel Method to Evaluate Ribosomal Performance in Cell-Free Protein Synthesis Systems

Noémie Kempf[1,†], Cristina Remes[1,¶,†], Ralph Ledesch[1], Tina Züchner[1], Henning Höfig[1,2], Ilona Ritter[1], Alexandros Katranidis[1,*] and Jörg Fitter[1,2,*]

[1]Institute of Complex Systems ICS-5, Forschungszentrum Jülich, 52428 Jülich, Germany,

[2]1. Physikalisches Institut (IA), RWTH Aachen, 52062 Aachen, Germany

[¶]Present address: Department of Mitochondrial Biology, Max Planck Institute for Biology of Ageing, 50931 Cologne, Germany

[†] These authors contributed equally to this work.

*Correspondence and requests for materials should be addressed to A.K. (email: a.katranidis@fz-juelich.de) or J.F. (email: j.fitter@fz-juelich.de)

Supplementary Text

Addition of Kasugamycin to the CFPS reaction

Kasugamycin (Ksg) is an antibiotic originally isolated from *Streptomyces kasugaensis* that blocks translation initiation by preventing the association of the ribosomal subunits, but it has no effect on translating or stalled 70S ribosomes[25-27]. The CFPS reactions in presence or in absence of 4 mM Ksg were performed as previously described, with 500 nM of ribosomes, 5.5 nM of linear plasmid (construct with SecM AP or SecMstr AP) and 5 nM of AS-tmRNA. Ksg was added 15 minutes after starting the reaction and the concentration of synthesized ${}^{SecM}_{lin}GFPem$ or ${}^{SecMstr}_{lin}GFPem$ was measured after 3h (Supplementary Fig. S3).

As expected, in the case of the highly efficient SecMstr AP construct, we observed that addition of Ksg 15 min after starting the CFPS reaction did not affect the final concentration of ${}^{SecMstr}_{lin}GFPem$, clearly indicating that the ribosomes are effectively stalled and unable to synthesize a second protein.

On the contrary, in the case of the less efficient SecM AP construct, addition of Ksg to the CFPS reaction after 15 min lead to a significant decrease of the final concentration of ${}^{SecM}_{lin}GFPem$. But surprisingly, the concentration of ${}^{SecM}_{lin}GFPem$ was not similar to the concentration of ${}^{SecMstr}_{lin}GFPem$. Instead, approximately half of the active ribosomes seemed to start a second cycle of synthesis within the first 15 min of the CFPS reaction. Nevertheless, addition of Ksg at an earlier time point (after 5 min) managed to fully inhibit the re-initiation leading to a concentration of ${}^{SecM}_{lin}GFPem$ similar to the one of ${}^{SecMstr}_{lin}GFPem$ (Supplementary Fig. S3, red bar). With these measurements we clearly confirm that the stalling efficiency of the SecM AP is much lower than the one of the SecMstr AP.

FCS measurements

To confirm the stalling efficiency of SecM and SecMstr arrest peptides (APs), we employed Fluorescence Correlation Spectroscopy (FCS) and compared the diffusion coefficients of GFPem synthesized with both APs, with the diffusion coefficients of free GFPem and non-specifically labeled control ribosomes (Supplementary Fig. S4). Since the diffusion of ribosomes is significantly slower than the diffusion of free GFPem, we were able to evaluate the stalling efficiency from the correlation curves of our samples. To this aim, the linear constructs of GFPem containing SecM (${}^{SecM}_{lin}GFPem$) and SecMstr (${}^{SecMstr}_{lin}GFPem$) were synthesized during two hours using CFPS kit, and their diffusion immediately measured.

In agreement with our co-precipitation results, the contribution of the large amount of freely diffusing protein led to a correlation curve for ${}^{SecM}_{lin}GFPem$ (Supplementary Fig. S4, red) much more comparable to the correlation curve of free GFPem than to labeled ribosomes. Interestingly, even though the correlation curve of ${}^{SecMstr}_{lin}GFPem$ was shifted to slower diffusion time (Supplementary

Fig. S4, blue), indicating that the stalling efficiency is increased, it was still not as it would be expected due to the effective stalling, as slow as ribosomes.

In addition, we noticed that both ${}^{SecM}_{lin}GFPem$ and ${}^{SecMstr}_{lin}GFPem$ FCS curves exhibited a microsecond timescale component (Supplementary Fig. S4, red and blue curves). It appeared to be proportional to the quantity of bound GFPem, but completely absent when free GFPem and 70S ribosomes were measured. Further anisotropy investigations indicated that this phenomenon appears as a consequence of a restricted rotation of the GFPem bound to the ribosome (data not shown).

It was previously reported that GFPem exhibits a very fast initial photobleaching component[42], which could bias our FCS analysis by reducing the emission time of the GFPem in the detection volume and lead to the conclusion that ${}^{SecM}_{lin}GFPem$ and ${}^{SecMstr}_{lin}GFPem$ diffused apparently faster than expected. In an attempt to overcome the bleaching of GFPem, we performed power series measurements with low excitation intensities for both ${}^{SecM}_{lin}GFPem$ and ${}^{SecMstr}_{lin}GFPem$ samples (Supplementary Fig. S4). Thus, the diffusion coefficient could be extrapolated to zero laser intensity (D_0) and the obtained value was then effectively corrected for photo-bleaching. From the corrected diffusion coefficients D_0, we could clearly show that the stalling efficiency of ${}^{SecMstr}_{lin}GFPem$ is significantly increased in comparison to the ${}^{SecM}_{lin}GFPem$ thus confirming the previous co-precipitation data. In addition, ribosome-like diffusion was observed after centrifugation regardless of the construct used. This indicates efficient separation of free and bound GFPem by centrifugation as well as physically still bound ribosome-nascent chain complexes after centrifugation. For mixed populations (samples which were not centrifuged) FCS cannot be employed to quantify the fraction values of bound and non-bound GFPs reliably. Hence the TCCD method was used to achieve this goal.

TCCD control measurement

The lower value of the stalling efficiency found with the TCCD method results from the presence of a few non-coinciding bursts in the blue detection channel. To find the origin of these bursts we separately measured with TCCD each element of the reaction mix: the buffer, the CFPS components and the ribosomes solution diluted for single-molecule measurements. From these measurements, blue bursts only appeared in the labeled ribosome solution, even though no GFPem was present since no reaction was performed. Surprisingly, these bursts did not coincide with the red bursts of the bioCAN^{Cy5} (Supplementary Table S1) indicating that they are not coming from the ribosomes themselves. Traces of fluorescent components could have been present as a result of the isolation procedure. Furthermore, results from TCCD measurements after a reaction performed in the same ribosome solution indicated that 70 blue bursts out of 79 (88%) were coinciding with a red burst from a bioCAN^{Cy5}. By subtracting the 6 background bursts, we then obtained 70 blue coinciding bursts out of 73 (96%), in better agreement with co-precipitation results.

conditions	Total blue bursts	non-coinciding blue bursts
buffer	0	0
CFPS components	0	0
ribosome solution	6	6
reaction mix	79	9

Supplementary Table S1. Results of the TCCD control measurements. Each value results from the average of three acquisitions of 5 minutes.

Supplementary Figures

```
1      MVSKGEELFTGVVPILVELDGDVNGHKFSVSGEGEGDATYGKLTLKFICT

51     TGKLPVPWPTLVTTLTYGVQCFARYPDHMKQHDFFKSAMPEGYVQERTIF

101    FKDDGNYKTRAEVKFEGDTLVNRIELKGIDFKEDGNILGHKLEYNYNSHK

151    VYITADKQKNGIKVNFKTRHNIEDGSVQLADHYQQNTPIGDGPVLLPDNH

201    YLSTQSALSKDPNEKRDHMVLLEFVTAAGITLGMDELYKLEGGGSGGGSG

                                FST--------------
251    GGSGGGSGGGSGGGGSGGGGSFSTPVWISQAQGIRAGPKLDPAANKARKE
                                FSTPVWIWWWPRIRGPP

301    AELAAATAEQ*
```

Supplementary Fig. S1. Amino acid sequence of the constructs. Primary sequence of the three constructs used in this study. The constructs differ only in the sequence of the arrest peptide. The gene of GFPem is highlighted green, the Gly/Ser linker blue and the stop codon red. The SecM arrest sequence is highlighted dark grey, the SecMstr arrest sequence light grey and the control, missing the last 14 amino acids, is not highlighted. The red amino acids in SecMstr depict the difference between the two arrest peptides.

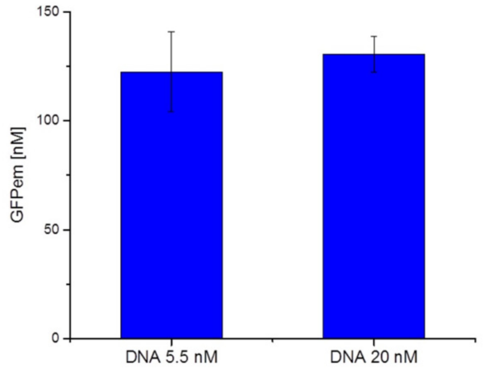

Supplementary Fig. S2. Non-limiting concentration of plasmid DNA for CFPS. Productivity of bioCAN ribosomes expressing ${}^{\mathrm{SecMstr}}_{\mathrm{lin}}\mathrm{GFPem}$, after addition of 5.5 nM or 20 nM of plasmid DNA to the CFPS system. The unaltered concentration of synthesized GFPem strongly suggests that the used conditions (5.5 nM) are non-limiting. Measurements were done after 3 h of reaction. The experiment was carried out in triplicates (technical replications).

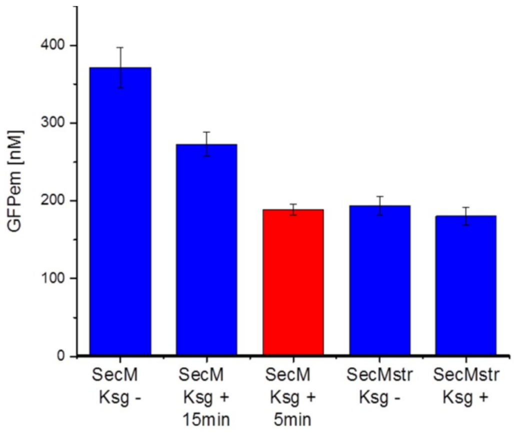

Supplementary Fig. S3. Effect of Kasugamycin on ribosomal productivity. Productivity of bioCAN ribosomes expressing $^{SecM}_{lin}GFPem$ and $^{SecMstr}_{lin}GFPem$, with and without addition of 4 mM Ksg 15 min after the beginning of the CFPS reaction. The constant concentration of synthesized $^{SecMstr}_{lin}GFPem$ upon addition of Ksg confirms the high stalling efficiency of the SecMstr AP. In the case of the SecM AP, addition of Ksg after 15 min led to a decrease of the $^{SecM}_{lin}GFPem$ concentration. Nevertheless, despite the decrease, half of the ribosomes seemed to already start a second cycle of translation in this time window. However, an earlier addition of Ksg (after 5 min) totally inhibited re-initiation of translation, leading to a concentration of $^{SecM}_{lin}GFPem$ similar to the concentration of $^{SecMstr}_{lin}GFPem$ (red bar). Measurements were done after 3 h of reaction. The experiment was carried out in triplicates (technical replications).

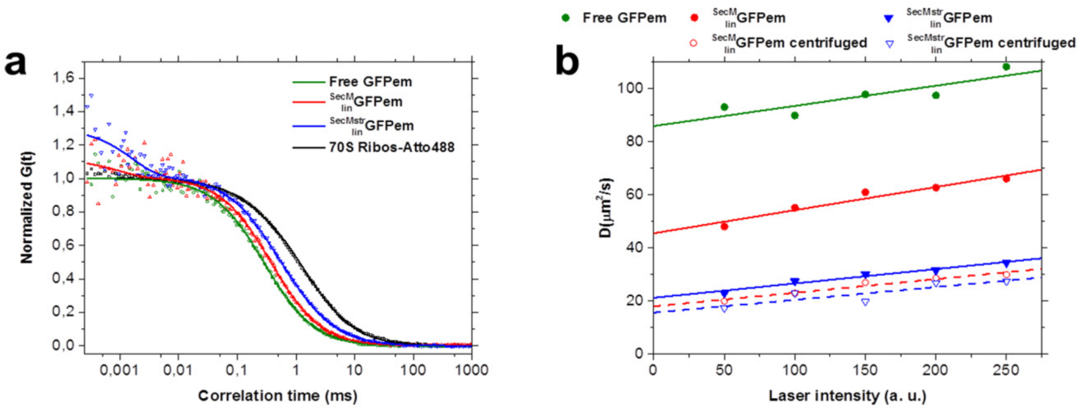

Supplementary Fig. S4. FCS Data. (a) FCS curves after 2 h CFPS reaction for ${}^{\mathrm{SecM}}_{\mathrm{lin}}\mathrm{GFPem}$ and ${}^{\mathrm{SecMstr}}_{\mathrm{lin}}\mathrm{GFPem}$ as compared to free GFPem and 70S ribosomes labeled with Atto488. Measurements were done at an intensity of 250 a.u. (~2μW). **(b)** Diffusion coefficients from FCS at different excitation intensities after 2 h CFPS reaction for ${}^{\mathrm{SecM}}_{\mathrm{lin}}\mathrm{GFPem}$ and ${}^{\mathrm{SecMstr}}_{\mathrm{lin}}\mathrm{GFPem}$ with and without sucrose precipitation. Diffusion coefficients D_0 extrapolated to zero laser intensity, where photobleaching effects no longer bias the obtained values[51]. As the fraction of bound GFPem increases the diffusion coefficient decreases. After centrifugation, ribosome-like diffusion is observed for all/both constructs.

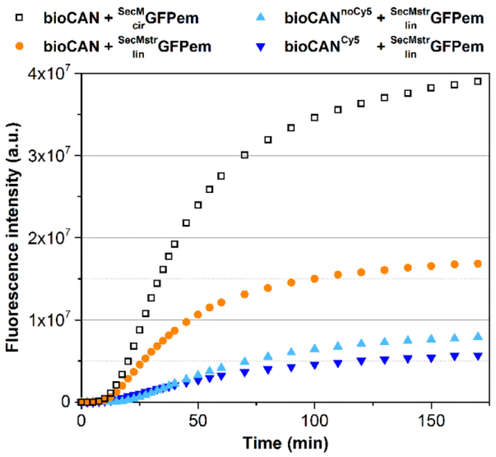

Supplementary Fig. S5. Productivity of bioCAN ribosomes expressing different constructs. Productivity (≈activity) of bioCAN ribosomes expressing ${}^{\mathrm{SecMstr}}_{\mathrm{lin}}\mathrm{GFPem}$ (efficient stalling, only one productive cycle) before labeling, after labeling with Cy5 and undergoing the labeling treatment without Cy5. The decrease after labeling is mostly due to the treatment and to a lesser extent depends on the fluorophore. Corresponding productivity of bioCAN ribosomes expressing ${}^{\mathrm{SecM}}_{\mathrm{cir}}\mathrm{GFPem}$ (almost no stalling) before labeling is given for comparison (representing the upper productivity limit of the CFPS system).

Construct	Mode	Fluorescence Intensity [a.u.]	Number of productive cycles
${}^{SecMstr}_{lin}GFPem$	batch	2.33682 x 10^7	1
${}^{SecM}_{cir}GFPem$	batch	4.14827 x 10^7	1.8
${}^{SecM}_{cir}GFPem$	CECF	3.65259 x 10^8	15.6

Supplementary Table S2. Productivity and number of productive cycles of bioCAN ribosomes under different CFPS modes. Productivity of bioCAN ribosomes expressing ${}^{SecM}_{cir}GFPem$ (almost no stalling) in a batch mode (after 2.5 h reaction) and in continuous exchange (CECF) mode (after 24 h). The number of productive cycles could be derived by comparing to the productivity (≈activity) of bioCAN ribosomes expressing ${}^{SecMstr}_{lin}GFPem$ (efficient stalling, only one productive cycle) in a batch mode (after 2.5 h reaction).
